# The President’s Malaria Initiative contributed to reducing malaria burden in sub-Saharan Africa between 2004 and 2014: Evidence from generalized estimating equation analysis

**DOI:** 10.1371/journal.pone.0217103

**Published:** 2019-05-24

**Authors:** Yazoume Ye, Dwomoh Duah

**Affiliations:** 1 ICF, Rockville, Maryland, United States of America; 2 University of Ghana, School of Public Health, Department of Biostatistics, Accra, Ghana; Instituto Rene Rachou, BRAZIL

## Abstract

The President’s Malaria Initiative (PMI) launched in 2005 as a key player in malaria prevention and treatment in sub-Saharan Africa (SSA). Several country-specific evaluations have demonstrated great progress in reducing under-five mortality associated with scaling up malaria interventions in PMI priority countries. Documentation of PMI’s specific contributions was limited, until the publication of Jakubowski, et al., which used difference-in-difference analysis to show a higher reduction of under-five mortality in PMI-supported countries than in others. To generate more evidence, this study used rigorous statistical analyses to assess the reduction in mortality attributable to PMI support. The study used generalized estimating equations and a series of matching procedures to evaluate the impact of PMI on under-five mortality and on population coverage of insecticide-treated nets (ITNs), indoor residual spraying (IRS), and artemisinin-based combination therapy (ACT) in SSA. The analyses used country-level secondary data and controlled for several country-level characteristics assumed to influence outcome measures of interest, PMI program participation, or both. The Mahalanobis distance metric, with 1:1 nearest neighbor matching adjusting for bias in population size in the particular country, showed a reduction in under-five mortality by approximately 12 per 1,000 live births (95% Confidence Interval [CI]: 20.6–3.1; p = 0.012). There were statistically significant increases in the population coverage of ITNs, IRS, and ACTs in PMI countries over the implementation period. ITN use in the population was 0.23% higher (95% CI average treatment effect on the treated: 0.17–0.30; p<0.001) in PMI-recipient countries than in non-PMI countries. The findings show that PMI contributed significantly to increasing the coverage of malaria control interventions and reducing under-five mortality in SSA.

## Introduction

Many government and international organizations, including the United States, have established reducing mortality among children under the age of five to the barest minimum as their primary goal. To that end, significant investments have been made to improve health and nutrition programs. Over the past decade, the U.S. Government, through the President’s Malaria Initiative (PMI), has invested significant funds in the health sector, particularly in sub-Saharan Africa (SSA), contributing to an expansion of key malaria prevention and treatment interventions with the potential to significantly reduce malaria burden [1–3].

Despite the increase in spending from PMI, little is known about the impact attributable to this initiative. In recent years, several country-specific impact evaluations have been conducted in PMI-supported countries, using the plausibility argument, to assess the change in all-cause child mortality associated with expansion of malaria interventions over the past decade. These evaluations documented significant progress in most of the countries [4–7]. However, the design of the evaluation could not measure the specific contribution of PMI to changes observed.

To bridge this gap, Jakubowski, et al.[1] used modified Poisson regression analysis with the difference-in-differences (DID) model to demonstrate that PMI support in countries was associated with the reduction in the annual risk of children under five between 2004 and 2014. The use of DID with fixed effect allows time-invariant country-specific characteristics to be correlated with the other measured independent variables, accounting for potential endogeneity. The authors used robust statistical analytic techniques; however, there are some challenges with their methods. The authors relied on the assumption that in standard DID estimation, the group composition does not change systematically over time. The population structure in the control (non-PMI) and intervention (PMI) countries could change from one survey to another within the same country between 2004 and 2014, creating a selection bias. Current methods to account for these selection biases in DID models are inadequate [8].

To address this problem, matching methods highlight areas of the distribution of covariates, in which the treatment and control groups lack sufficient overlap, such that the resulting treatment- effect estimates would rely on extrapolation. Matching with regression-based model adjustments on the matched samples reduces bias from covariate differences. Furthermore, regression analysis on the matched samples could adjust for the small remaining differences and increase the efficiency of estimating impact. Using a series of nationally representative data from 32 countries in SSA between 2004 and 2014, this study used generalized estimating equations (GEE) regression and matching inferential methods to estimate the impact of PMI support on mortality among children under five, comparing countries receiving PMI support to those not receiving PMI support. The study hypothesized that both GEE and the matching technique would also result in a positive impact of PMI support, as found by Jakubowski, et al.[1]

## Methods

### Considerations in the choice of the statistic approach

To quantify the impact of PMI support on the reduction of under-five mortality (U5M), one must account for several other factors that could contribute to change in U5M in SSA. Controlling and comparing factors at one time point before the rollout of PMI support and assuming that all other factors would remain unchanged between PMI-recipient and non-PMI recipient countries could lead to biased impact estimates. These country-level characteristics vary over time, and their effect should be examined accordingly. This systematic difference between PMI- and non-PMI- recipient countries makes the two groups different, and changes in the outcome variable may not be attributable to the program. This study takes into account time-variant, country-level characteristics that could be associated with U5M. These factors could influence either the possibility of receiving PMI intervention, U5M, or both (confounders). These factors include gross national income (GNI) per capita based on purchasing power parity (PPP), health expenditure, government effectiveness index, political stability index, corruption index, rule of law, voice and accountability, and other large health funding sources.

### Countries included in the analysis

**Fig 1:**
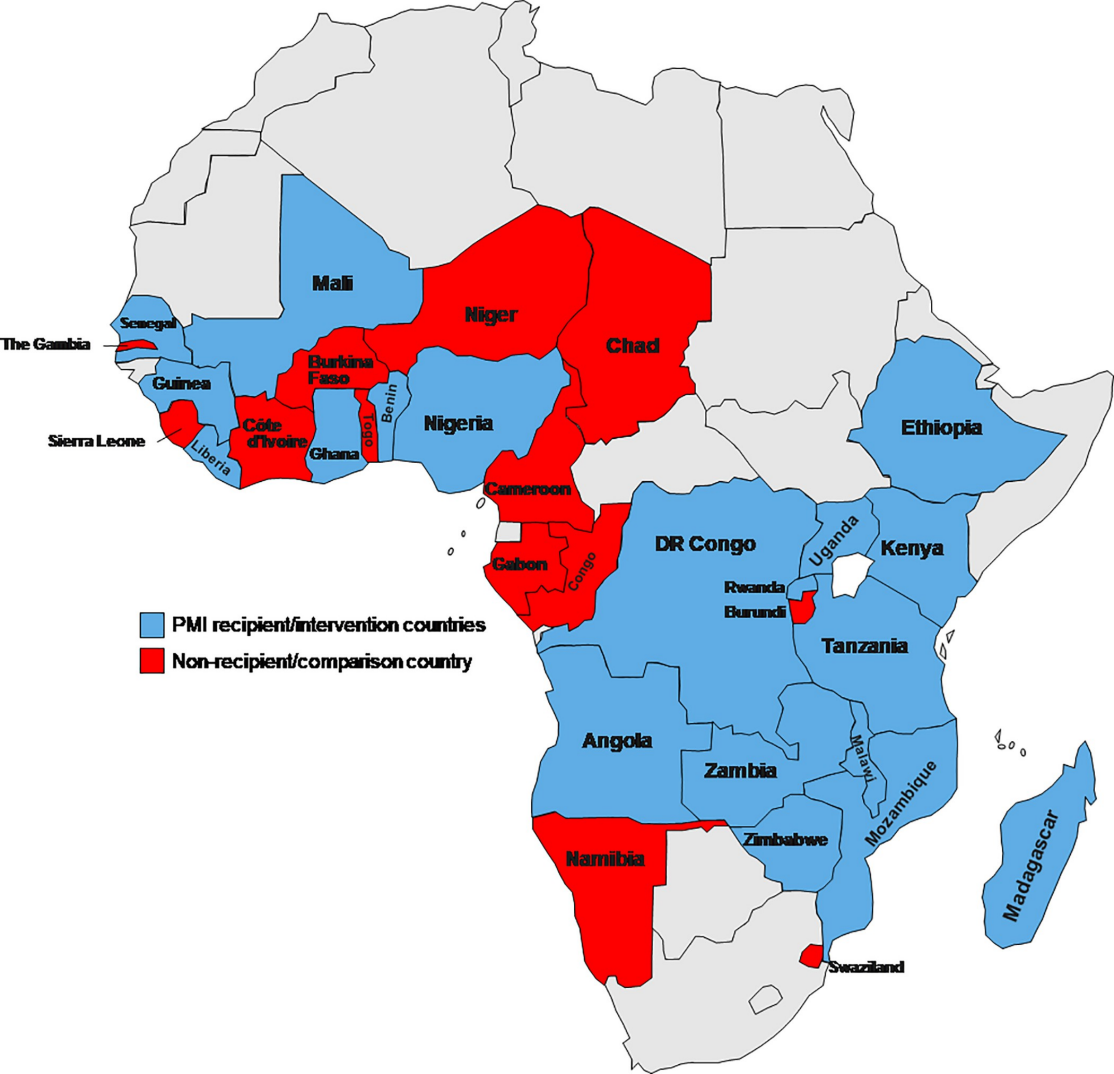
Geographical distribution of countries included in the study. **PMI recipient/intervention countries (Year start):** Angola (2006), Uganda (2006), Republic of Tanzania (2006),Malawi (2007), Mozambique (2007), Rwanda (2007), Senegal (2007), Benin (2008), Ethiopia (2008), Ghana (2008), Kenya (2008), Liberia (2008), Madagascar (2008), Mali (2008), Zambia (2008), Democratic Republic of the Congo (2011), Guinea (2011), Nigeria (2011), Zimbabwe (2011).

This study involved 32 countries from SSA. Nineteen countries received the intervention (PMI), and 13 countries served as the non-PMI-recipient (control) countries in this study. Of the 19 countries receiving the intervention, three (Angola, Tanzania, and Uganda) have received the initiative since its full implementation in 2006. The Democratic Republic of the Congo, Guinea, Nigeria, and Zimbabwe were the last four countries to have received the intervention in 2011 (Fig 1).

### Intervention and program variables of interest

The President Malaria Initiative, launched in 2005, began operation in 2006, with the goal of reducing malaria-related morbidity and mortality by 50% across 15 high-burden countries in SSA through a rapid scale-up of four proven and highly effective malaria prevention and treatment measures. These effective interventions were insecticide-treated nets (ITNs), indoor residual spraying (IRS), accurate diagnosis and prompt treatment with artemisinin-based combination therapies (ACTs), and intermittent preventive treatment of pregnant women. Data on beneficiary countries were obtained from the Eleventh Annual Report to Congress, submitted in April 2017. For details on PMI’s intervention, see PMI website [9].

The selection of PMI countries is based on several criteria. In this study, the authors assumed that country selection was based on country-year characteristics, such as being a recipient of other development assistance for health, neonatal mortality rate, government effectiveness index, voice and accountability, regulatory quality, rule of law, political stability, control of corruption index, average *Plasmodium falciparum* rate in children ages 2–10, GNI per capita based on PPP, total population, total health expenditure, and rural population between 2004 and 2014. The study also assumed that the propensity of a country to receive the intervention is independent of the education level of the child’s mother, mother’s age, child’s age, socioeconomic status, and other household-level characteristics.

PMI support was measured as a binary indicator, coded as “0” if the PMI program did not provide funding to a given country in a given year and coded as “1” if funding was provided. PMI was also measured as a continuous variable to evaluate the impact of the program intensity (per capita disbursements in a given country and year).

### Outcome measures

#### Primary outcome measure

All-cause U5M rates (U5MRs) were obtained from the United Nations Inter-agency Group for Child Mortality Estimation (United Nations Children’s Foundation, World Health Organization, World Bank, United Nations Department of Economic and Social Affairs Population Division) at www.childmortality.org for 32 countries in SSA between 2004 and 2014 for PMI- and non-PMI-recipient countries. The U5MR was defined as the probability per 1,000 that a newborn will die before reaching age five, if subject to age-specific mortality rates of the specified year.

#### Secondary outcome measures

Other outcome measures of interest in this study were population coverage of malaria prevention and treatment. These estimates were obtained from the Malaria Atlas Project [10] between 2004 and 2014 for both PMI- and non-PMI-recipient countries. These secondary outcome measures, which also serve as explanatory variables in the U5MR models, are as follows:

- Population coverage of ITNs: Proportion of people who slept under an ITN on any given night each year
- Population coverage of IRS: Proportion of the population protected by IRS
- Population coverage of ACTs: Proportion of fever cases in children under five receiving ACTs

#### Other competing health interventions

The study used a continuous measure variable called “Other development assistants for health minus PMI” (ODAH-PMI) and controlled for it in the analysis. The ODAH-PMI is the amount of financial and in-kind support tracked from the source to the recipient country, region, or health focus area. This variable sums up all other health interventions, such as the U.S. President’s Emergency Fund for AIDS Relief, the Global Fund to Fight AIDS, Tuberculosis and Malaria, domestic interventions, and all other health disbursements. Data were obtained from the Institute for Health Metrics and Evaluation [11].

#### Other country-level explanatory variables

Other country-level explanatory variables are the following: average *Plasmodium falciparum* rate in children ages 2–10, GNI per capita based on purchasing power parity (PPP), total population, health expenditure, rural population, government effectiveness index, voice and accountability, regulatory quality, rule of law, political stability—no violence, and control of corruption index between 2004 and 2014. Data were obtained from the World Bank website [12].

### Statistical analysis

The authors used graphical tools for longitudinal/panel data analysis to explore the trend of U5MR and other parameters of interest between PMI- and non-PMI-recipient countries. Two types of analysis were performed. To assess the association of PMI, ITNs, ACTs, IRS, and U5MR, the authors first fitted a series of GEE. GEE from the Gaussian distribution were used to account for the correlated structure of the data set, [13,14] because observations within a country are more correlated than those between countries. The authors then performed a series of different matching techniques, including nearest neighbor matching based on propensity score and Mahalanobis distance metric measures, and Mahalanobis with propensity score matching (PSM) caliper and subclassification on the propensity score, to establish cause-effect relationships among PMI, ITNs, ACTs, IRS, and U5MR. Statistical significance was considered at alpha = 0.05 using two-tailed tests. All statistical analysis were performed with Stata version 15 (StataCorp, College Station, Texas, USA).

#### Estimating PMI participation (propensity score model accounting for clustering)

The authors first estimated the propensity for country *i* to participate in or receive PMI support in year *j*, which is unknown. They considered certain time-varying factors that could influence the probability of a country receiving PMI support, U5MR, or both. The authors incorporated clustered structure (country-level) in PSM to account for bias from unmeasured cluster-level confounders. The propensity score estimation based on the probit regression model considered the following factors: effective governance, rule of law, regulatory quality, voice and accountability, corruption index, political stability, GNI, recipient of other health intervention, domestic health expenditure, and proportion of rural population. These factors were assumed to have a direct effect on U5MR, the propensity of a country to receive the PMI intervention, or both [15–18].

Population coverage of ITNs, ACTs, IRS, and average *Plasmodium falciparum* rate in children ages 2–10 were not included in the propensity score model, because these variables may have been affected by the treatment variable of interest (PMI).

#### Coarsened exact matching

To achieve consistency and evaluate the robustness of our impact estimate, Coarsened Exact Matching (CEM) [19] was used. This is a relatively new method for improving the estimation of causal effects by reducing imbalance in covariates—in this case, between PMI-recipient countries and nonrecipient countries. Countries were not selected randomly to receive PMI support, so the authors controlled for a set of pretreatment variables using the CEM algorithm. CEM bounds the degree of model dependence and causal effect estimation error by ex-ante user choice. It is monotonic imbalance bounding, which means that reducing the maximum imbalance on one variable—between PMI- and non-PMI-recipient countries—has no effect on other variables.

#### Assessing common support and diagnosing matches from PSM

Numerical and graphical diagnoses were used to observe the common support of the distribution of propensity score between PMI- and non-PMI-recipient countries. The authors compared the multidimensional histograms and Kernel density plots of the covariates in the matched PMI and non-PMI countries. In this study, the authors assumed that the standardized differences greater than 10 percent in absolute value indicate a serious imbalance in the covariate of interest between PMI- recipient and nonrecipient countries [20]. The standardized difference *d* is given as follows:

$$d=\frac{\left({\bar{X}}_{PMI\;recipient}-{\bar{X}}_{PMI\;non-recipient}\right)}{\sqrt{\frac{S_{PMI}^{2}+S_{NonPMI}^{2}}{2}}}\times 100\%$$

In using CEM, the authors assessed the overall imbalance of covariates given by the $L_{1}$ statistic between PMI- and non-PMI-recipient countries. This imbalance measure was introduced [21] as a comprehensive measure of global imbalance.

#### Sensitivity analysis of GEE

**GEE:**This study tested the robustness of the impact estimates of PMI support on U5MR with a series of sensitivity analyses. The authors assessed whether the results from the GEE analysis were robust to the type of model chosen, by estimating the effect of PMI on U5MR using Poisson, negative binomial, and gamma distribution. Sensitivity analyses of different specifications of the GEE intra-cluster correlation matrix were also performed based on autoregressive with lag 1 wave.

**Matching procedures:** Analyses of sensitivity to the ignorability assumption under PSM were performed. The authors calculated the bounds [22] for average PMI effects on the treated in the presence of unobserved heterogeneity (hidden bias) between PMI-recipient and -nonrecipient countries. Because different matching techniques produce slightly different results (impact estimates), Mahalanobis distance metric matching, Mahalanobis matching within propensity score calipers, subclassification, k:1, and nearest neighbor matching were performed to confirm the robustness of the impact estimate. In addition, because the intensity of aid disbursement varies among recipient countries over time, the authors estimated the amount received U5MR function in which the PMI-recipient countries might take on a continuum of values to ascertain whether similar results could be replicated.

## Results

### Comparing covariates between PMI- and non-PMI-recipient countries

The estimated U5MR per 1,000 live births between 2004 and 2014 was approximately 94 for PMI countries and approximately 108 for non-PMI-recipient countries, and the difference was statistically significant (*p*<0.001). PMI-recipient countries had relatively higher population coverage of ITNs, ACTs, and IRS. With the exceptions of corruption index, government effectiveness, and political stability (*p*>0.05), there was a statistically significant difference in all other covariates between PMI-recipient and -nonrecipient countries over time. Neonatal mortality rate, *Plasmodium falciparum* transmission rate for children ages 2–10, proportion of rural population, and domestic health expenditure were found to be higher in the PMI-recipient countries than in non-PMI-recipient countries (2004–2014). Governance indices, such as voice and accountability, regulatory quality, and rule of law, were found to be better over the years in PMI-recipient countries. Other health development assistants (million US$) were found to be significantly higher in PMI-recipient countries over the years, as shown in Table 1.

**Table 1:** Comparing covariates between PMI recipient and nonrecipient countries (2004–2014).

Covariates	Non-PMI-recipient countries (n = 13)	PMI-recipient countries (n = 19)	Statistical significance test Unadjusted p-value estimates from GEE with Gamma and Gaussian models (Robust SE)
	*median*,*iqr* (mean±*sd*)	*median*,*iqr* (mean±*sd*)	
ITN (x100%):scale of 0–1	0.116, 0.316	0.31, 0.263	< 0.0001***
IRS (x100%):scale of 0–1	0.000, 0.005	0.057, 0.098	0.019*
ACT (x100%): scale of 0–1	0.024, 0.062	0.092, 0.138	< 0.001***
Neonatal mortality rate: scale of 0–100%	32.10±7.45	30.46±8.65	< 0.001***^ǂ^
Population size	9891790, 13600000	21200000, 24300000	< 0.001***
*Plasmodium falciparum* rate in ages 2–10: scale of 0–1	0.235, 0.351	0.177, 0.281	< 0.001***
Other health development assistant (million US$)	7479.038, 10955.365	9449.636, 13473.930	< 0.001***
Domestic health expenditure (million US$)	88.116, 107.78	98.835, 68.547	< 0.002**
Gross national income (PPP)	1425, 1950	1760, 1240	<0.001***
Rural population (%)	62.942, 26.503	66.388, 17.551	< 0.001***
Government effectiveness index: -2.5 (weak) to 2.5 (strong)	-0.900, 0.570	-0.580, 0.530	0.120
Voice and accountability: -2.5 (weak) to 2.5 (strong)	-0.801± 0.535	-0.459± 0.548	0.019*^ǂ^
Regulatory quality: -2.5 (weak) to 2.5 (strong)	-0.775± 0.473	-0.563±0.430	< 0.001***^ǂ^
Rule of law: -2.5 (weak) to 2.5 (strong)	-0.869±0.500	-0.658± 0.4311	0.047*^ǂ^
Political stability: -2.5 (weak) to 2.5 (strong)	-0.580±0.8296	-0.640±0.7429	0.497^ǂ^
Control of corruption index: -2.5 (weak) to 2.5 (strong)	-0.910, 0.550	-0.620, 0.510	0.085
Number of observation	**352**		

***p<0.001

**p<0.01

*p<0.05

GEE: generalized estimating equations, SE: Standard Error, iqr: Interquartile range

ǂ: generalized estimating equations from Gaussian distribution with identity link. Positively skewed outcomes variables were assessed with Gamma distribution.

### Results on matching procedure

**Fig 2:**
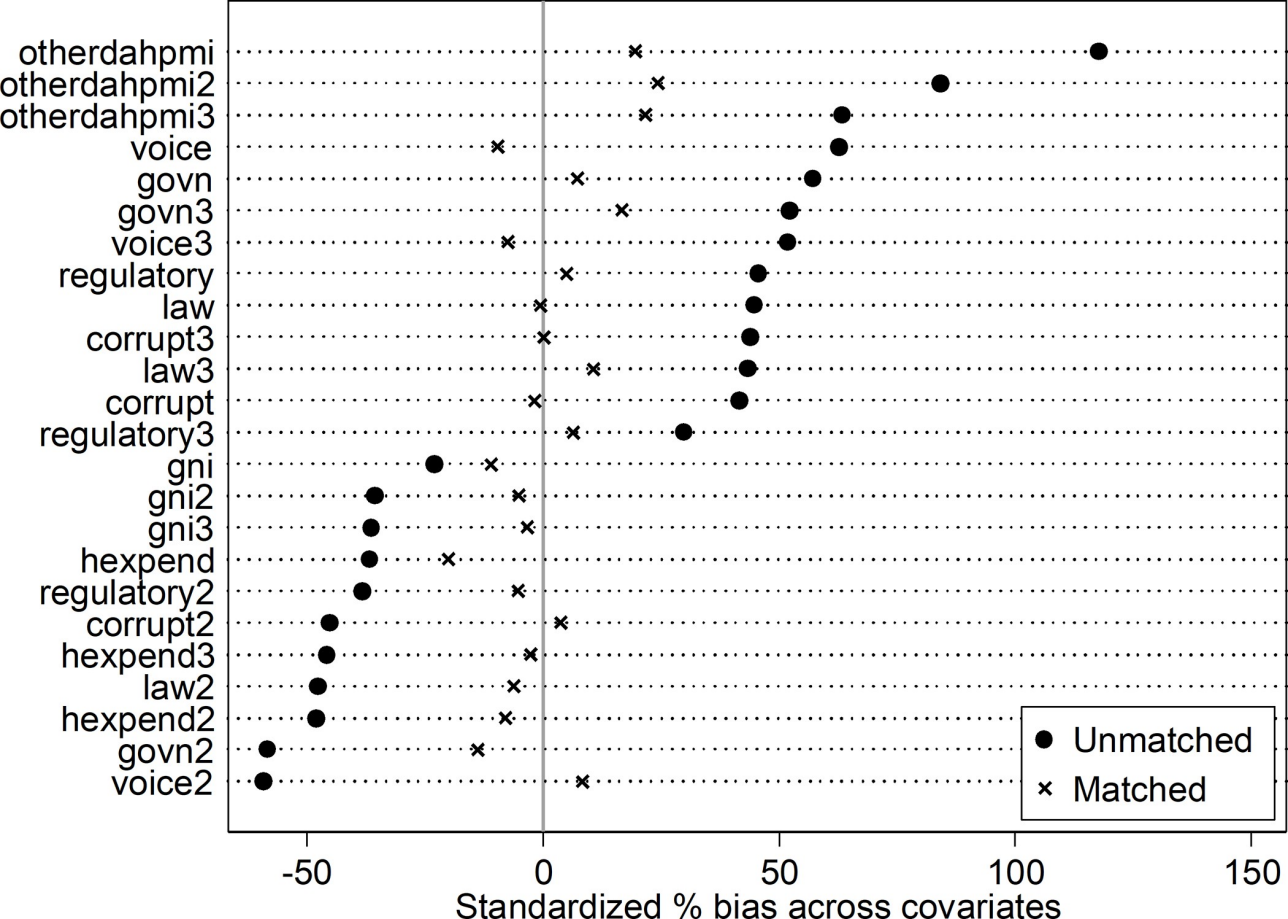
Covariate balance between PMI- and non-PMI-recipient countries: Standardized percentage bias across covariates. **Abbreviation**: hexpend = Domestic health expenditure, gni = gross national income, otherdahpmi = Other development health assistant minus PMI, govn: Effective governance, voice: Voice and accountability, regulatory: Regulatory quality; index number 2 and 3 represent 2^nd^ and 3^rd^ order polynomial terms, respectively.

The propensity score histogram by PMI status of countries in Fig 2 showed moderate overlap of the propensity scores, with a good control match for each PMI-recipient country. This indicates that few PMI countries did not find a good match (lack of common support). The diagnostic graph assessment of the covariate balance between PMI- and non-PMI-recipient countries showed that standardized percentage bias between the two groups reduced drastically after matching for all covariates used in the propensity score model (Fig 2). For instance, bias in GNI between the two groups reduced from -23.1% to -11.1%, resulting in an absolute percentage reduction in bias of 52%. The bias in voice and accountability index between PMI- and non-PMI-recipient countries reduced to 84.7% after matching. Between the two groups after matching, bias in other health development assistance was reduced by 83.5%, bias in domestic health expenditure was reduced by 45.1%, bias in effective governance was reduced by 87.3%, and bias in corruption was reduced by 95.6%. The overall imbalance in corruption before matching given by the *L1* statistic, introduced by Blackwell, et al., [19] was 27.2% and was reduced to 11.1% after matching (Table 2).

**Table 2:** Standardized differences between PMI-recipient and non-PMI-recipient countries.

	Before matching				After matching				
Variable	PMI recipient	Non-PMI recipient	Bias (%)	p-value	PMI recipient	Non-PMI recipient	Bias(%)	p-value	*% rABS(Bias)*
GNI	2053.4	2622.6	-23.1	0.041	2053.4	1501.2	22.4	<0.0001	3.0
GNI^2^	5.8×10^6^	1.7×10^7^	-35.7	0.002	5.8×10^6^	2.9×10^6^	8.7	<0.0001	75.6
GNI^3^	2.1×10^10^	1.9×10^11^	-36.4	0.002	2.1×10^10^	1.7×10^9^	3.0	<0.0001	97.6
VOICE	-0.46	-0.80	62.60	<0.0001	-0.46	-0.48	3.50	0.759	94.40
VOICE^2^	0.51	0.93	-59.30	<0.0001	0.51	0.52	-1.10	0.920	98.1
VOICE^3^	-0.57	-1.10	51.90	<0.0001	-0.57	-0.59	2.30	0.822	95.50
DEXPEND	114.06	163.46	-36.80	0.001	114.06	92.27	16.20	0.001	55.90
DEXPEND^2^	16144.00	59362.00	-48.20	<0.0001	16144.00	11936.00	4.70	0.043	90.30
DEXPEND^3^	2.8×10^6^	3.1×10^7^	-45.90	<0.0001	2.8×10^6^	2.1×10^6^	1.00	0.305	97.80
REG^1^	-0.57	-0.77	45.60	<0.0001	-0.57	-0.68	25.90	0.026	43.40
REG^2^	0.50	0.82	-38.30	0.001	0.50	0.70	-24.00	0.036	37.40
REG^3^	-0.57	-1.03	29.80	0.007	-0.57	-0.90	21.20	0.054	29.00
LAW	-0.66	-0.87	44.70	<0.0001	-0.66	-0.68	4.90	0.661	89.00
LAW^2^	0.62	1.00	-47.80	<0.0001	0.62	0.69	-9.40	0.397	80.30
LAW^3^	-0.70	-1.27	43.40	<0.0001	-0.70	-0.85	11.30	0.304	73.90
EGOV	-0.68	-0.92	57.10	<0.0001	-0.68	-0.74	14.60	0.181	74.50
EGOV^2^	0.63	1.03	-58.40	<0.0001	0.63	0.70	-11.40	0.294	80.40
EGOV^3^	-0.67	-1.24	52.10	<0.0001	-0.67	-0.78	9.80	0.353	81.30
COR	-0.63	-0.81	41.50	<0.0001	-0.63	-0.68	12.10	0.259	71.00
COR^2^	0.59	0.84	-45.20	<0.0001	0.59	0.60	-3.00	0.773	93.30
COR^3^	-0.56	-0.91	43.80	<0.0001	-0.56	-0.58	2.20	0.828	95.00
ODAH	3.8×10^8^	1.1×10^8^	117.80	<0.0001	3.8×10^8^	2.2×10^8^	70.00	<0.0001	40.50
ODAH^2^	2.4×10^17^	2.7×10^16^	84.10	<0.0001	2.4×10^17^	8.4×10^16^	61.70	<0.0001	26.6
ODAH^3^	1.9×10^26^	1.5×10^25^	63.40	<0.0001	1.9×10^26^	5.5×10^25^	48.90	<0.0001	22.8
**Median bias**	**45.8**				**7.4**				
**Rubin’s B**	**147.0**				**109.1**				
**Rubin’s R**	**0.27**				**1.33**				

Abbreviations: Rubin’s B: The absolute standardized difference of the means of the linear index of the propensity score in the treated (PMI recipient) and (matched) non-treated group (non-PMI-recipient countries); Rubin's R: The ratio of treated (PMI-recipient countries) to (matched) non-treated variances of the propensity score index. Rubin (2001) recommends that B be less than 25 and that R be between 0.5 and 2 for the samples to be considered sufficiently balanced. GNI: gross national income (Purchasing Power Parity), VOICE: Voice and accountability, DEXPEND: Domestic Health Expenditure, REG: Regulatory Quality, EGOV: Effective Governance, LAW: Rule of Law, COR: Corruption index, ODAH: Other Development Health Assistance minus PMI. Subscript number represent number of iterations of the variable, % rABS(Bias): Percentage reduction in absolute bias; P-value notation: p<0.05 statistically significant. Note: Biases in observed country-level covariates were reduced greatly between PMI and non-PMI-recipient countries when rural population and political stability were eliminated from the propensity score model.

### Impact estimates of PMI in reducing U5MR and increasing population coverage of ITNs, IRS, and ACTs

Adjusting for key covariates (ITNs, IRS, and ACTs), the Mahalanobis distance metric matching within propensity score calipers showed that U5MR reduced by 11 per 1,000 live births (95% CI ATET: 18.7–2.3; p = 0.012) in the PMI countries compared to the non-PMI countries. Mahalanobis distance metric with 1:1 nearest neighbor matching adjusting further for bias in population size and the particular country resulted in the reduction of U5MR by approximately 12 per 1,000 live births (95% CI ATET: 20.6–3.1; p = 0.012). There was a statistically significant increase in the population coverage of ITNs, IRS, and ACTs for PMI countries over the implementation period. Population coverage of ITN use increased by 0.23% (95% CI ATET: 0.17–0.30; p<0.001) for PMI-recipient countries compared to non-PMI countries. The impact of PMI became weightier with the use of 2:1 nearest neighbor matching based on Mahalanobis distance metric as the number of under-five deaths reduced to approximately 13 per 1,000 live births (95% CI ATET: 0.17–0.30; p = 0.002) in the PMI countries. Similar patterns were observed for population coverage of ITNs, IRS, and ACTs (Table 3).

**Table 3:** Impact of PMI and sensitivity analysis on primary and secondary outcome measures of interest using different matching procedures.

Matching procedures and sensitivity analysis	U5MR per 1000 live birth	ITN (%)	IRS (%)	ACT (%)
	ATET (95% CI)	ATET (95% CI)	ATET (95% CI)	ATET (95% CI)
**Matching procedures**				
Mahalanobis metric matching on covariates within propensity score caliper: Variables used in estimating Mahalanobis metric for U5MR includes IRS, ITN, and ACT	-10.50 (-18.66, -2.33); p = 0.012			
Mahalanobis distance metric with nearest neighbor matching: 1:1: Biases adjusted for population size and country	-11.86 (-20.61, -3.10); p = 0.008	0.23 (0.17, 0.30) P<0.001	0.08 (0.06,0.11) P<0.001	0.11 (0.08, 0.13); P<0.001
Mahalanobis distance metric with nearest neighbor matching: 2:1: Biases adjusted for population size and country	-12.65(-20.48, -4.817); p = 0.002	0.23 (0.18, 0.29); p<0.001	0.08 (0.06,0.10); p<0.001	0.10 (0.08, 0.13) P<0.001
**Sensitivity Analysis**				
Coarsened Exact Matching with propensity score: Checking imbalance on corruption	-9.60 (-20.11, 0.89); p = 0.073	0.13 (0.08,0.18), p<0.001	0.07 (0.02, 0.12); p = 0.005	0.07 (0.04,0.10); p<0.001
Coarsened Exact Matching	-13.83 (-21.98, -5.68); p = 0.001	0.16 (0.11,0.21); p<0.001	0.04 (0.00, 0.08); p = 0.034	0.05 (0.02,0.08); p<0.001
Subclassification on propensity score: Five subclasses	-17.34 (-24.45, -6.50); p<0.05	0.18 (0.12, 0.21); p<0.05	0.04 (-0.01, 0.07); p>0.05	0.08 (0.07; 0.10) p<0.05

U5MR: Under-five mortality rate; Population coverage of insecticide-treated nets (ITNs): Proportion of people who slept under insecticide-treated bednet on any given night each year; Population coverage of indoor residual spraying (IRS): Proportion of the population protected by indoor spraying of insecticides (IRS); Population coverage of artemisinin-based combination therapy (ACTs): Proportion of fever cases in under-five-year-olds receiving ACTs. Variance in the treatment group is much larger than that in the control group, smaller calipers were necessary as indicated by Rosenbaum and Rubin (1985b). The authors therefore used a caliper of 0.25 standard deviations of the linear propensity score: CI: Confidence interval based on robust standard error, ATET: Average PMI effect on recipient countries. For Mahalanobis with K: 1 Nearest neighbor matching, biased was adjusted for population size and country, PSM: Propensity score matching; CEM: Coarsened Exact Matching: Estimating the sample average treatment on the treated (the SATT), P-value notation: p<0.05 statistically significant.

## Discussion

This study evaluated the impact of PMI on reducing U5MR and increasing population coverage of ITNs, IRS, and ACTs using population-averaged regression models for panel data analysis and matching procedures, which serve as one of several methods of estimating the causal effect of interventions using observational data. The use of matching procedures replicated a randomized experiment as closely as possible, by obtaining treatment (PMI-recipient countries) and control groups (non-PMI-recipient countries) with similar country-level covariate distributions to reduce bias from these measured covariates.

PMI was found to have statistically significant impact on the reductions in all-cause mortality rates among children under five and in the increase in population coverage of ITNs, IRS, and ACTs. These findings were consistent with the results obtained by Jakubowski, et al., [1] who used a DID analytic technique to determine significant statistical associations between PMI support and U5MR, ITNs, ACTs, and IRS. It is worth emphasizing that ITN use, ACTs, and IRS are all in the causal pathway of U5MR in SSA. This shows that increasing population coverage of these interventions through PMI support reduces U5MR. Several other studies have shown the effectiveness of these intermediate outcomes in increasing population coverage of IRS, ITNs, and ACTs, as was the case in this study. Eisele, et al., [23] based on two systematic literature reviews in *Plasmodium falciparum* endemic settings, have demonstrated the effect of the coverage of ITNs and IRS on preventing malaria-attributable mortality in children ages 1–59 months. Furthermore, Gimnig, et al. [24] also used an analytic technique similar to this study (propensity scores with regression models) to evaluate the effect of IRS programs on malaria-related outcomes and arrived at findings that were similar to our results.

Although sensitivity analysis was conducted to critically evaluate the robustness of our results, there may be several unmeasured variables that could systematically affect U5MR and the use of ITNs, IRS, and ACTs. Country-specific interventions that were not measured and controlled for in the analysis could affect the results of our findings, especially when these unknown factors affect PMI and comparison countries in different ways. Apart from the usual government interventions and funding partners’ support in terms of health spending that are usually captured in a national budget, several other nongovernmental organizations have supported initiatives to control malaria. These initiatives may not be known, and the data required to measure how they influence health outcomes are not readily available, particularly considering the need for cross-country comparisons. Furthermore, differences in country-specific malaria interventions and the proportion of children under five who are covered by these interventions could bias our results, because the lack of reliable data prevented this study from controlling for them. This study did not examine the spill-over effect of PMI, and future research should explore whether the investments made by PMI in recipient countries improved other health and non-health outcomes, such as improved immunization coverage, health infrastructure, educational attainment, socioeconomic status, access to and use of health care services, maternal and newborn care, and general quality of life. Unintended consequences, such as corruption and sale of other bed nets by private individuals in the health sector, could also be investigated.

That notwithstanding, this study contributes to the expanse of literature detailing the efficacy of health interventions. The findings provide additional evidence of significant decreases in child mortality rates after the introduction of PMI. There is an inverse relationship between PMI funding and reduction of all-cause U5M. In addition, PMI has resulted in higher population coverage of ITNs, IRS, and ACTs, which is in the causal pathway of all-cause U5M. Global players need to make a concerted effort to sustain these gains and strive for malaria elimination, in line with the Global Technical Strategy 2016–2030. PMI’s role will continue to be critical, and the recent expansion of PMI’s support to four additional countries will accelerate progress toward eliminating malaria in SSA.

## Supporting information

S1 Table Data on coverage of artimisinin-based combination theraphies. (XLS)

S2 Table Data on coverage of indoor residual spray. (XLS)

S3 Table Data on coverage of insecticide treated nets. (XLS)

S4 Table Data on access to sanitation. (XLS)

S5 Table Data on mortality among under five children. (XLS)

S6 Table Data on additional country level co-variates. (XLS)
